# Family Behavioral Repertoires and Family Interaction Influence the Adaptive Behaviors of Individuals With *Hikikomori*

**DOI:** 10.3389/fpsyt.2019.00977

**Published:** 2020-01-16

**Authors:** Shunsuke Nonaka, Hironori Shimada, Motohiro Sakai

**Affiliations:** ^1^ School of Child Psychology, Tokyo Future University, Tokyo, Japan; ^2^ Faculty of Human Sciences, Waseda University, Saitama, Japan; ^3^ Faculty of Education, University of Miyazaki, Miyazaki, Japan

**Keywords:** adaptive behaviors, behavioral repertoires, Hikikomori, family, family interaction, parents, social withdrawal

## Abstract

**Background:** Family support is key in the initial stages of psychological support for individuals with *hikikomori*. However, it remains necessary to confirm the relationship between families’ cognitive behavioral factors and the severity of hikikomori to understand ways of improving hikikomori. We examined the influences of family behavioral repertoires for coping with hikikomori and family interaction on the adaptive behaviors of individuals with hikikomori. We employed a control group to examine whether the influence of these adaptive behaviors was unique to families of individuals with hikikomori.

**Methods:** We asked 185 parents of individuals with hikikomori (hikikomori group) and 460 parents of individuals with no experience of hikikomori (control group) to complete the Family Behavioral Repertoire Scale for coping with hikikomori (FBS-H), the Family Interaction Scale for Hikikomori (FIS-H), and the Adaptive Behaviors Scale for Hikikomori (ABS-H). Using the subscales of the ABS-H as the dependent variables, we conducted hierarchical multiple regression analyses wherein family behavioral repertoire was added in Step 1, experience frequency and cognition of contingency were added in Step 2 as control values, family interaction was added in Step 3, and the interaction terms were added in Step 4.

**Results:** The ABS-H total and subscale scores were significantly lower in the hikikomori group than in the control group. The social participation subscale showed the largest difference, while the family subscale showed the smallest. In the hikikomori group, we observed a significant adjusted *R* *^2^* for the family and value subscales (Step 1). The *ΔR* ^2^ in Step 3 was significant for the interaction and family subscales of the ABS-H. In the control group, significant adjusted *R* ^2^ values were found for all ABS-H subscales in Step 1, but the *ΔR* ^2^ in Step 3 was not significant for any subscales.

**Conclusion:** Family-related cognitive behavioral factors, such as family behavioral repertoire and family interaction, appear to relate to improvement in hikikomori. Of course, these findings warrant further investigation because we did not examine the longitudinal, causal relations between these variables. In the future, we might also test the effect of family support interventions that target families’ behavioral repertoire and family interaction.

## Introduction

*Hikikomori* (prolonged social withdrawal) was defined by the Ministry of Health, Labour and Welfare’s research group as a phenomenon with the characteristic features of avoidance of social interactions—such as avoidance of school attendance, working, and socializing outside one’s home, and staying at home almost every day (save for solitary outings)—for more than half a year (1). A study on the epidemiology of hikikomori in a community-based population aged 20–49 years (*n* = 1,660) in Japan revealed that 1.2% had experienced the phenomenon in their lifetime (2). Although hikikomori was initially regarded as a distinctively Japanese phenomenon, several studies have reported on the prevalence of individuals with hikikomori in other countries, such as Australia, France, India, Korea, and the USA [e.g., (3–6)].

Many individuals with hikikomori have genuine physical and psychosocial difficulties (2, 6–12). Kondo et al. (7) reported that 80.3% of individuals with hikikomori who utilized the services of mental health welfare centers had a diagnosed psychiatric disorder, such as a mood, anxiety, personality, or developmental disorder. Additionally, Nonaka and Sakai (8) indicated that individuals with hikikomori have significantly lower quality of life than do those who have never experienced hikikomori. Nakagaito et al. (9) reported that many long-term individuals with hikikomori (i.e., those who have had hikikomori for more than 15 years) displayed not only psychological problems but also severe physical problems, such as nutritional disorders and voice disturbances. Accordingly, we believe that hikikomori, in many cases, is not merely laziness, and we speculate that the number of people who show this phenomenon globally will increase in the future (13, 14).

Due to the characteristics of this condition, the best avenue for assessing and supporting individuals with hikikomori is indirectly *via* the family, particularly in the initial stage. Family members themselves also often face considerable difficulty in caring for these individuals (15) and tend to be the people who first begin seeking help for the condition; in only 7% of cases are the initial help seekers individuals with hikikomori themselves (16). Therefore, the most important characteristic of psychosocial support for hikikomori is that therapists cannot access people with hikikomori directly (1).

There are various family support approaches for hikikomori cases, such as family therapy, psychoanalysis, self-help groups, and cognitive behavior therapy (CBT) (1, 17, 18). Some studies have reported the effects of CBT (17, 19); however, there are very few researches on the family support of hikikomori cases. The improvement process has not always been clarified, and studies on CBT have been limited to examining the overall effect of CBT on family; there has been surprisingly little research on the extent to which the specific cognitive behavioral factors of the family targeted by CBT can influence hikikomori. Clarifying this influence will be useful for improving the effect of family support. Family behavioral repertoire (13) and family interaction (14) are the family’s cognitive behavioral factors that influence the improvement of hikikomori. In theory, when the family acquires a behavioral repertoire, it is possible to have a basis for using the behavioral repertoire according to the situation of communicating with individuals with hikikomori. As a result, it is speculated that the family interaction will function more positively.

Two particularly important cognitive behavioral factors related to family support are the family behavioral repertoire for coping with hikikomori (20) and family interaction (21). Several studies note that family relationships and parenting styles do not necessarily affect the “expression” of hikikomori. For example, when comparing individuals with hikikomori and those without experience of hikikomori, we found that the influence of the family behavioral repertoire and family interaction are not strongly related to the expression of hikikomori (20, 21). Additionally, Umeda et al. (22), who examined the influence of childhood family environments on the hikikomori experience, reported that childrearing styles do not significantly differ between hikikomori and non-hikikomori groups. Taken together, these findings suggest that the influence of the family might play little role in the “expression” of hikikomori.

However, it would be important to clarify the influence of family-related cognitive behavioral factors on the “improvement” process of hikikomori, given the importance of family support in the initial stage of the condition. So far, no studies have examined this influence. Hikikomori means a decrease in social interaction behavior, which is considered an adaptive behavior (23, 24); accordingly, the less adaptive behavior is performed, the more severe the hikikomori is expected to be. Therefore, the primary purpose of this study was to determine the influence of the “family behavioral repertoire” for coping with hikikomori and “family interaction” on the adaptive behaviors of individuals with hikikomori, according to a hypothesis model. To this end, we used hierarchical regression models to explore the associations of these two family-related cognitive behavioral factors with adaptive behaviors. We assumed a hypothesis model that family interaction is unlikely to be functional unless the family has acquired a sufficient behavioral repertoire. Thus, we could make a hypothesis that the family’s behavioral repertoire influences adaptive behaviors of hikikomori, and family interaction strengthens the influence. Therefore, in step 1, we added the family’s behavioral repertoire. Also, as factors influencing family interaction, there are the “frequency of experience of family interaction scenes (the frequency of experiencing specific parent–child interaction scenes in daily life)” and cognition of contingency [which refers to the ability of the family to recognize the results of their own communication (21)]. Therefore, in step 2, scene experience frequency and cognition of contingency were added as the control variables, and family interaction was added in step 3. Furthermore, family cognitive behavioral factors such as behavioral repertoire, frequency of experience of family interaction scenes, cognition of contingency, and family interaction might interactively influence the adaptive behaviors of individuals with hikikomori. For example, it is expected that the behavioral repertoire would have a stronger influence on adaptive behaviors when the cognition of contingency is high. Therefore, the interaction terms of these cognitive behavioral factors were also examined in hierarchical multiple regression analysis.

Additionally, in a secondary purpose of this study, we assumed that individuals with hikikomori are less influenced by environments outside the family than non-hikikomori cases. Therefore, people with hikikomori would be more influenced by their families than people without hikikomori, even when both groups have similar influences from outside the family. Thus, we predicted that the family interaction would have a relatively stronger influence on the adaptive behaviors of individuals with hikikomori than on the adaptive behaviors of people with no experience of hikikomori. Although we could not compare this prediction directly, to investigate it indirectly, we recruited a control group of people without hikikomori.

## Methods

### Data Collection

There is plenty of research on Japanese hikikomori cases [e.g., (25)], which largely shows that the Japanese cultural background influences the expression and maintenance of hikikomori (26). Thus, we collected all data from Japanese families in this study. We recruited two samples for this study: family associations of individuals with hikikomori and a web-based normative sample. The family members of individuals with hikikomori all belonged to family associations and support centers in Japan, and were recruited through these associations/centers. The family associations and support centers were initially asked to participate in the investigation, after which we sent questionnaires by mail. Staff at these institutions subsequently asked family members to complete the questionnaires anonymously and return them by mail or in person. The web-based sample consisted of parents of individuals aged 16–49 years from a large-scale web-based research panel in Japan. All these individuals voluntarily agreed to participate. Individuals were free to withdraw from participation at any time. During the analysis, the groups were matched in terms of relationship with the child (father or mother) and the child’s gender and age, to facilitate descriptive comparison and clarify whether the influence of family cognitive behavioral factors on children’s adaptive behavior was peculiar to hikikomori cases or not. Between the two samples, we matched the proportion of participants according to relationship with the child, and child’s gender and age group (by 5 years). We classified participants into two groups: parents of individuals with no experience of hikikomori (control group) and parents of individuals who have experienced hikikomori (hikikomori group). To be eligible to participate in this study, participants had to respond to all items regarding their own age and gender as well as the age, gender, and duration of hikikomori of the individuals with hikikomori.

### Measures

#### Demographics

Participants reported their age and relationship with the child (father or mother), the child’s general characteristics (gender, age), and the child’s experience of hikikomori (1), both currently and in the past.

#### Adaptive Behaviors Scale for Hikikomori (ABS-H)

Because hikikomori shows a state in which social interaction behavior is restricted, the adaptive behavior of the individuals with hikikomori is social interaction behavior in many cases. The social interaction behavior of the individuals with hikikomori includes communicating with family or non-family members, social behavior toward their goals, and working or attending school (23). The ABS-H is a parent-rated measure of the social interaction behaviors of individuals with hikikomori. It consists of four subscales: interaction, family, value (behaviors that match the values of individuals with hikikomori), and social participation inside and outside the home (23). The ABS-H comprises 26 items in total, which require participants to assess the frequency of children’s adaptive behaviors on a 4-point scale ranging from 0 (almost never) to 3 (almost always). Higher scores on the ABS-H indicate more adaptive behaviors. The ABS-H has adequate reliability, criterion-related validity, discriminant validity, and construct validity (23). Cronbach’s alpha showed similar values to a previous study (23), with the hikikomori group α = 0.94, control group α = 0.95.

#### Family Behavioral Repertoire Scale for Coping With Hikikomori (FBS-H)

This scale comprises 25 items assessing the behavioral repertoire of the family members of individuals with hikikomori. It comprises four subscales: cooperative (i.e., “Talk to the child with a kind expression”), assertive (i.e., “Try inviting the child gently if there is anything that the child is interested in”), self-control [i.e., “Unaware of what kind of emotion I am feeling when I contact a child” (Reversed items)], and cheerful (i.e., “Talk with a bright expression matching the mood when talking about fun”). Participants rated the items on a 4-point scale ranging from 1 (not applicable) to 4 (applicable). Higher scores indicate a greater family behavioral repertoire for coping with hikikomori. The FBS-H has satisfactory reliability, convergent validity, and discriminant validity (20). The greater the family behavior repertoire, the more the parents have acquired different ways of coping with hikikomori (e.g., sometimes cooperative, assertive, or cheerful). Cronbach’s alpha showed similar values to a previous study (20), with the hikikomori group α = 0.89, control group α = 0.90.

#### Family Interaction Scale for Hikikomori (FIS-H)

The FIS-H measures the experience frequency (i.e., “Are told ‘Good morning’ by your son/daughter”), cognition of contingency (i.e., the ability of families to recognize the results of their own communication), and degree of family interaction in 12 family interaction scenes (**Appendix**). The items describe behaviors of parents in these situations and require parents to indicate whether an increase or decrease occurred in the responses of individuals with hikikomori after parents’ behavior. For example, to measure family interaction, we asked “In association with your son/daughter, how likely is this behavior to change?” (after presenting each family interaction scene). More specifically, based on operant conditioning theory, the FIS-H measures whether certain parental approaches to individuals with hikikomori are functional or nonfunctional in specific scenes (21). According to operant conditioning theory, if one’s own reaction reinforces the other’s behavior, the behavior increases and, if the reaction punishes the other’s behavior, the behavior decreases. The “cognition of contingency” indicates the ability to adequately recognize the relationship between another’s behavior and one’s own reaction. Thus, a high cognition of contingency indicates that it is easy for parents to predict the results of their communication. Participants rate the frequency of their child’s behaviors on a 5-point scale ranging from 1 (decreased) to 5 (increased). Higher scores indicate more functional family interaction. The FIS-H has sufficient reliability, convergent validity, and discriminant validity (21). Cronbach’s alpha showed similar values to previous study (21) with the hikikomori group α = 0.83, control group α = 0.86 in cognition of contingency, and the hikikomori group α = 0.80, control group α = 0.87 in family interaction.

### Data Analysis

All data were analyzed using R version 3.4.1 (27) with the “psych” (28) and “mice” (29) packages. Recently, researchers have recommended multiple imputation or maximum likelihood estimation as the best methods of handling missing values (30). We used multiple imputation to handle missing data at the item level of each scale. The results across 50 imputed data sets were combined. We used hierarchical multiple regression analysis to examine the influence of the family’s cognitive behavioral factors on the adaptive behaviors of individuals with hikikomori. Specifically, with the subscales of ABS-H as the dependent variables, we conducted separate regression analyses wherein the FBS-H was added in Step 1, the experience frequency and cognition of contingency subscales of the FIS-H were added in Step 2 as control values, the family interaction subscale was added in Step 3, and the interaction terms between FBS-H and FIS-H were added in Step 4. We centered the variables in the interaction terms to minimize the impact of multicollinearity.

### Ethical Consideration

The study was approved by the local research ethics committee of the institute to which the author(s) belong. We obtained informed consent before conducting the study. In consideration of individuals’ privacy, the study was carried out anonymously.

## Results

### Missing Data

The item-level missing data rates for the main variables were all less than 4% (0.00–3.41%). Overall, 851 records (1.40%) were missing out of a total of 60,630 records.

### Participants

**Figure 1:**
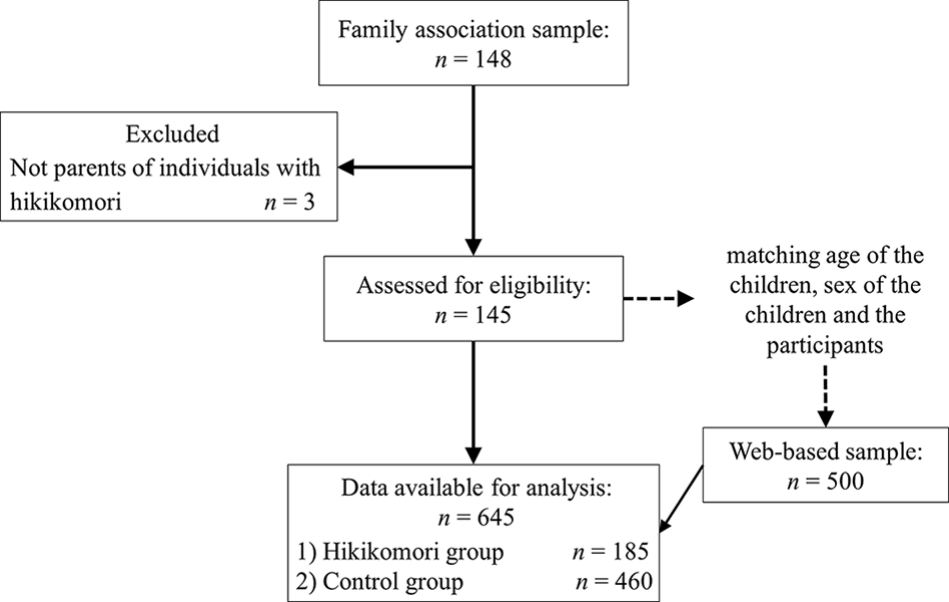
Participants**’** recruitment and follow-up flow.

We obtained 148 individuals from the family associations of individuals with hikikomori and 500 from the web-based panel (**Figure 1**). Participants who were not the parents of the child in question were excluded (**Table 1**).

**Table 1:** Demographic characteristics of participants.

	Hikikomori group		Control group	
Participants				
Father, n [%])	46	[24.87]	125	[27.13]
Age, M [*SD*]	63.10	[7.82]	60.49	[8.22]
Living with the child, *n* [%]	157	[84.87]	212	[46.09]
Children				
Male, *n* [%]	116	[84.06]	383	[83.26]
Age, *M* [*SD*]	32.55	[8.36]	32.13	[8.37]
Duration of hikikomori (month), *M* [*SD*]	109.79	[89.46]	-	
n	185		460	

Hikikomori group, parents of individuals with hikikomori; Control group, parents of individuals with no experience of hikikomori.

### Descriptive Statistics of Main Variables

**Table 2** shows the means and standard deviations, as well as group comparisons (including effect sizes), of the total and subscale scores of the ABS-H, FBS-H, and FIS-H. The ABS-H total and subscale scores were all significantly lower in the hikikomori group than in the control group. The social participation subscale showed the greatest difference and the family factor showed the smallest. There was no significant difference between the groups for the FBS-H total score. For the experience frequency subscale of the FIS-H, the control group had higher scores than the hikikomori group. However, the cognition of contingency and family interaction scores were higher in the hikikomori group than in the control group.

**Table 2:** Means, standard deviations, and effect sizes in each group.

	Hikikomori group		Control group		*ES*	*ES* 95% CI		*n*
	*M*	*SD*	*M*	*SD*		lower	upper	
ABS-H								
Interaction	14.11	(9.19)	30.23	(7.96)	1.94	1.73	2.14	633
Family	6.35	(3.46)	8.95	(2.50)	0.93	0.75	1.11	637
Value	4.95	(3.15)	8.64	(2.75)	1.29	1.10	1.48	635
Social	2.95	(3.57)	10.09	(2.01)	2.82	2.58	3.05	634
Total	28.18	(16.32)	57.91	(13.26)	2.11	1.89	2.32	622
FBS-H	76.94	(9.86)	75.94	(9.95)	0.10	−0.08	0.28	622
FIS-H								
Scene experience frequency	10.31	(5.67)	13.50	(4.25)	0.68	0.50	0.86	627
Cognition of contingency	45.34	(6.33)	43.27	(6.24)	0.33	0.15	0.51	618
Family interaction	42.90	(5.92)	41.76	(6.25)	0.18	0.00	0.37	615

Hikikomori group: parents of individuals with hikikomori; control group: parents of individuals with no experience of hikikomori; ABS-H, Adaptive Behaviors Scale for Hikikomori; FBS-H, Family Behavioral Repertoire Scale for Hikikomori; FIS-H, Family Interaction Scale for Hikikomori; ES, effect size (Hedge’s *g*).

### Influence of Family’s Behavioral Repertoires and Family Interaction on Hikikomori

We calculated variance inflation factor (VIF) to check for multicollinearity problems. Both the hikikomori (VIFs < 2.95) and control groups did not show large values (VIFs < 3.00), indicating no problem of multicollinearity. The results of the hierarchical multiple regression analyses in the hikikomori group (**Table 3**) revealed significant adjusted coefficients of determination for the family and value subscales in Step 1 (family: *R* ^2^ adj = 0.18, *β* = 0.42, *p* < 0.001; value: *R* ^2^ adj = 0.03, *β* = 0.18, *p* < 0.05). Furthermore, the *ΔR* ^2^ in Step 3 was significant for the interaction (*ΔR* ^2^ = 0.04, *β* = 0.21, *p* < 0.05) and family subscales (*ΔR* ^2^ = 0.05, *β* = 0.17, *p* < 0.05) of the ABS-H. However, the *ΔR* ^2^ in Step 4 was not significant for any of the ABS-H subscales.

**Table 3:** Results of hierarchical multiple regression analysis of hikikomori group.

	Interaction		Family		Value		Social participation	
	β		β		β		β	
Step 1								
FBS-H	0.14	†	0.42	***	0.18	*	0.02	
* R* ^2^ adj	0.02	†	0.18	***	0.03	*	0.00	
Step 2								
FBS-H	0.07		0.29	***	0.10		0.01	
FIS-H/S	0.32	***	0.48	***	0.31	***	0.16	†
FIS-H/C	−0.05		−0.01		−0.03		−0.12	
* R* ^2^ adj	0.12	***	0.43	***	0.13	***	0.03	*
* ΔR* ^2^	0.11	***	0.25	***	0.11	***	0.04	*
Step 3								
FBS-H	0.05		0.24	***	0.09		0.01	
FIS-H/S	0.36	***	0.56	***	0.38	***	0.18	*
FIS-H/C	−0.19	†	−0.11		−0.12		−0.21	†
FIS-H/I	0.21	*	0.17	*	0.11		0.04	
* R* ^2^ adj	0.14	***	0.48	***	0.16	***	0.03	
* ΔR* ^2^	0.04	*	0.05	*	0.04		0.01	
Step 4								
FBS-H	0.03		0.24	***	0.10		0.00	
FIS-H/S	0.39	***	0.55	***	0.39	***	0.14	
FIS-H/C	−0.20	†	−0.12		−0.12		−0.18	
FIS-H/I	0.20	†	0.19	*	0.11		0.04	
FBS-H×FIS-H/S	−0.05		−0.02		0.05		−0.01	
FBS-H×FIS-H/C	−0.02		−0.04		0.04		−0.10	
FBS-H×FIS-H/I	0.00		−0.03		−0.06		0.11	
FIS-H/S×FIS-H/C	−0.09		−0.01		0.00		0.08	
FIS-H/S×FIS-H/I	0.09		0.05		−0.01		0.04	
FIS-H/C×FIS-H/I	0.05		0.00		−0.01		0.03	
* R* ^2^ adj	0.12	**	0.47	***	0.13	***	0.02	
* ΔR* ^2^	0.01		0.01		0.01		0.03	

FBS-H, Family Behavioral Repertoire Scale for Coping with Hikikomori; FIS-H, Family Interaction Scale for Hikikomori; FIS-H/S, scene experience frequency; FIS-H/C, cognition of contingency; FIS-H/I, family interaction, ^†^p < 0.10, *p < 0.05, **p < 0.01, ***p < 0.001.

**Figure 2:**
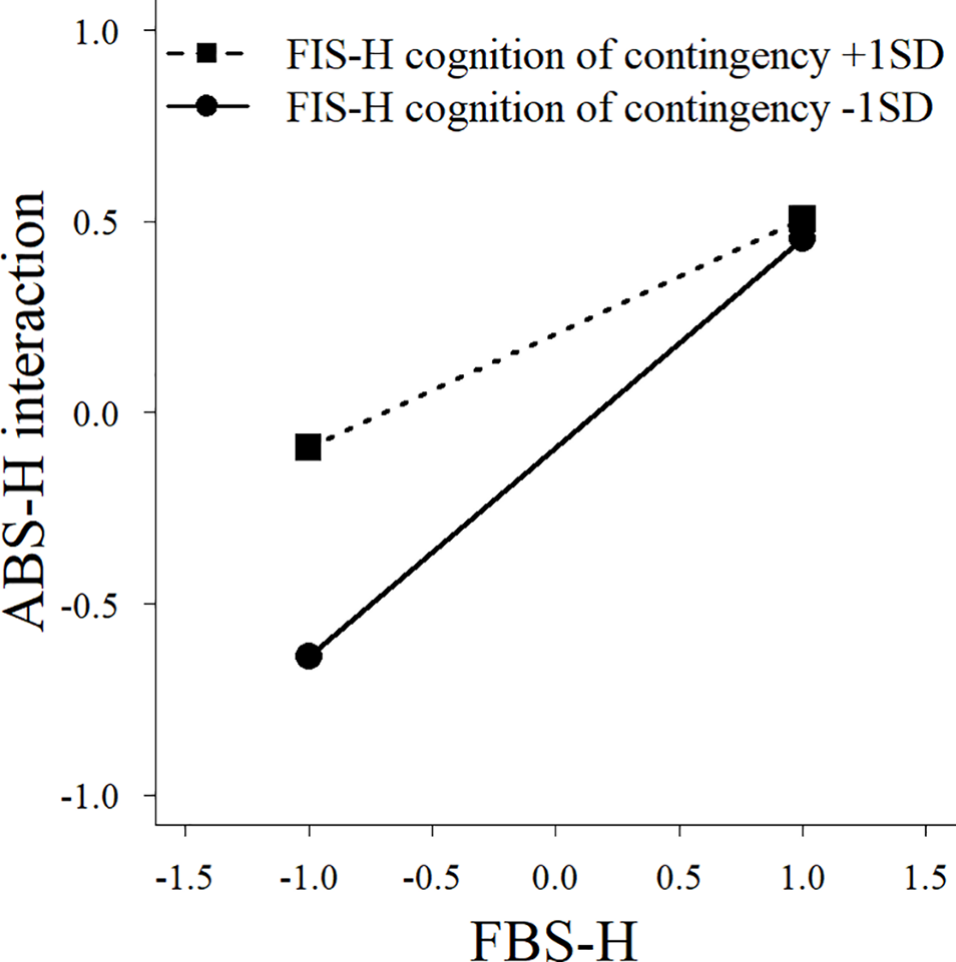
A simple slope analysis between family behavioral repertoire and cognition of contingency for the interaction factor of the Adaptive Behaviors Scale for Hikikomori among parents of individuals with no experience of hikikomori. FBS-H, Family Behavioral Repertoire Scale for Coping with Hikikomori; ABS-H, Adaptive Behaviors Scale for Hikikomori.

**Figure 3:**
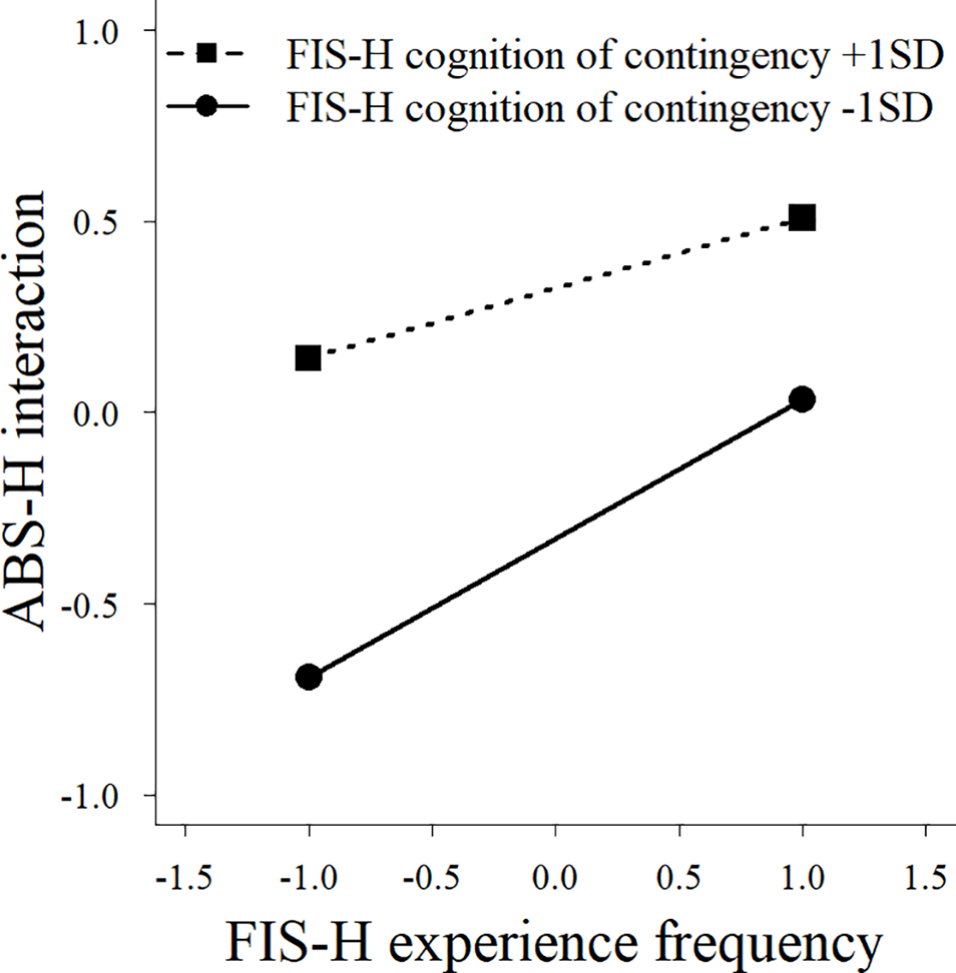
A simple slope analysis between family experience frequency and cognition of contingency for the interaction factor of the Adaptive Behaviors Scale for Hikikomori among parents of individuals with no experience of hikikomori. FIS-H, Family Interaction Scale for Hikikomori; ABS-H, Adaptive Behaviors Scale for Hikikomori.

**Figure 4:**
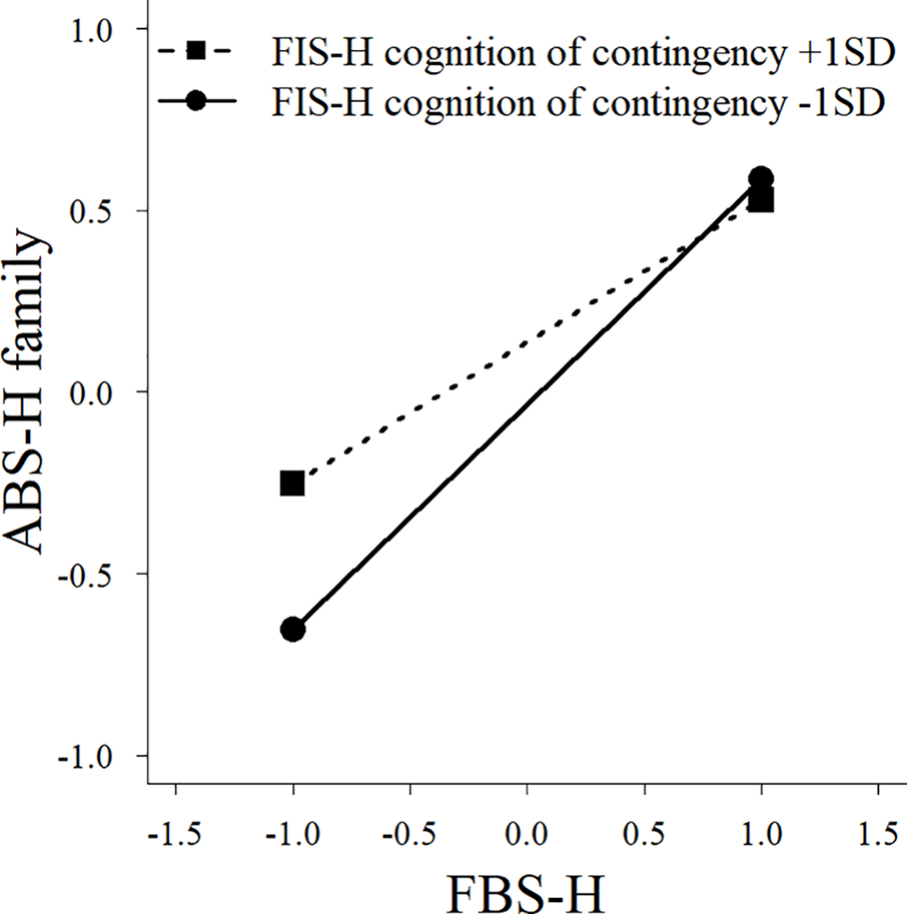
A simple slope analysis between family behavioral repertoire and cognition of contingency for the family factor of Adaptive Behaviors Scale for Hikikomori among parents of individuals with no experience of hikikomori. FBS-H, Family Behavioral Repertoire Scale for Coping with Hikikomori; ABS-H, Adaptive Behaviors Scale for Hikikomori.

The results of the hierarchical multiple regression analyses of the control group (**Table 4**) showed significant adjusted coefficients of determination for all ABS-H subscales in Step 1 (interaction: *R* ^2^ adj = 0.24, *β* = 0.50, *p* < 0.001; family: *R* ^2^ adj = 0.30, *β* = 0.55, *p* < 0.001; value: *R* ^2^ adj = 0.19, *β* = 0.44, *p* < 0.001; social participation: *R* ^2^ adj = 0.16, *β* = 0.40, *p* < 0.001). Although the *ΔR* ^2^ in Step 3 was not significant for any of the ABS-H subscales, in Step 4, it was significant for all subscales (interaction: *ΔR* ^2^ = 0.03, *p* = 0.001; family: *ΔR* ^2^ = 0.02, *p* = 0.03; value: *ΔR* ^2^ = 0.02, *p* = 0.04; social participation: *ΔR* ^2^ = 0.02, *p* = 0.04). We also observed significant interaction effects between family behavioral repertoire and cognition of contingency (**Figure 2**), and between experience frequency and cognition of contingency (**Figure 3**), on the interaction subscale of the ABS-H. Furthermore, there was a significant interaction effect of family behavioral repertoire and cognition of contingency on the family subscale (**Figure 4**). Simple slope analysis showed that the influence of family behavioral repertoire was greater when cognition of contingency was lower for the interaction subscale (high: *β* = 0.30, *p* < 0.001; low: *β* = 0.55, *p* < 0.001) and family subscale of the ABS-H (high: *β* = 0.18, *p* = 0.009; low: *β* = 0.36, *p* < 0.001). In addition, for the interaction subscale of the ABS-H, the influence of experience frequency was greater when the cognition of contingency was lower than when it was higher (high: *β* = 0.39, *p* = 0.009; low: *β* = 0.62, *p* < 0.001).

**Table 4:** The results of the hierarchical multiple regression analysis in the control group.

	Interaction		Family		Value		Social participation	
	β		β		β		β	
Step 1								
FBS-H	0.50	***	0.55	***	0.44	***	0.40	***
* R* ^2^ adj	0.24	***	0.30	***	0.19	***	0.16	***
Step 2								
FBS-H	0.39	***	0.47	***	0.35	***	0.31	***
FIS-H/S	0.22	***	0.25	***	0.19	***	−0.03	
FIS-H/C	0.14	**	0.08	†	0.12	**	0.21	***
* R* ^2^ adj	0.30	***	0.36	***	0.23	***	0.19	***
* ΔR* ^2^	0.06	***	0.06	***	0.04	***	0.04	***
Step 3								
FBS-H	0.39	***	0.46	***	0.34	***	0.30	***
FIS-H/S	0.22	***	0.25	***	0.19	***	−0.03	
FIS-H/C	0.11	*	0.03		0.08		0.16	**
FIS-H/I	0.05		0.07		0.08		0.08	
* R* ^2^ adj	0.30	***	0.36	***	0.23	***	0.20	***
* ΔR* ^2^	0.00		0.00		0.00		0.00	
Step 4								
FBS-H	0.37	***	0.43	***	0.31	***	0.29	***
FIS-H/S	0.21	***	0.26	***	0.20	***	0.02	
FIS-H/C	0.19	**	0.09		0.13	†	0.20	**
FIS-H/I	−0.02		0.06		0.07		0.08	
FBS-H×FIS-H/S	−0.01		−0.04		−0.07	†	0.07	†
FBS-H×FIS-H/C	−0.13	**	−0.11	*	−0.09	†	−0.10	†
FBS-H×FIS-H/I	−0.02		−0.02		0.00		−0.01	
FIS-H/S×FIS-H/C	−0.16	*	0.03		−0.01		−0.01	
FIS-H/S×FIS-H/I	0.12	†	0.03		0.10		0.07	
FIS-H/C×FIS-H/I	0.04		0.00		−0.02		−0.02	
* R* ^2^ adj	0.32	***	0.37	***	0.25	***	0.21	***
* ΔR* ^2^	0.03	**	0.02	*	0.02	*	0.02	*

FBS, Family Behavioral Repertoire Scale about coping with Hikikomori, FIS, Family Interaction Scale for Hikikomori; FIS/S Scenes experience frequency, FIS/C, Cognition of contingency, FIS/I, Family interaction, ^†^p < 0.10, *p < 0.05, **p < 0.01, ***p < 0.001.

## Discussion

The hikikomori group had lower scores for adaptive behaviors than the control group. Interestingly, the social participation subscale showed the largest difference, while the family subscale showed the smallest difference. These results support the findings of previous studies (23). Furthermore, family behavioral repertoire did not significantly differ between the groups. This result supports the findings of previous research (20). Although the experience frequency score of the family interaction scenes was higher in the control group than in the hikikomori group, the cognition of contingency and family interaction scores were higher in the hikikomori group. These findings also support previous studies (21).

The findings indicated that family behavioral repertoire is significantly associated with the adaptive behaviors of individuals with hikikomori, and that family interaction enhances this association. A similar tendency was observed for the interaction subscale of the ABS-H. We also found that family behavioral repertoire was associated with the value subscale in the hikikomori group. Taken together, the results indicate that family behavioral repertoire and family interactions, despite having no effect on social participation such as school attendance or work, may help promote the adaptive behaviors relating to social interaction with family or others, and the value of individuals with hikikomori. The fact that there was no association with social participation suggests that individual differences in social participation were large, and therefore inconsistent among people with hikikomori. Therefore, it may be necessary to account for such individual differences by providing not only indirect support such as family support (31–33) but also direct support.

In the control group, family behavioral repertoire was found to influence various adaptive behaviors in children, whereas family interaction did not appear to be associated with adaptive behaviors. This latter finding is potentially because those without experience of hikikomori have greater social interaction outside the family, and social interaction outside the family has a relatively strong effect on individuals. By contrast, the living environment of individuals with hikikomori is restricted to the home, meaning that individuals with hikikomori are likely to be more influenced by family interaction patterns. Accordingly, although this study did not compare the hikikomori and control groups directly, it seems necessary not only to extend the family behavioral repertoire of individuals with hikikomori, but to also help improve the functionality of their family interactions to increase the adaptive behaviors of individuals with hikikomori.

The interaction effects found in the control group indicate that family behavioral repertoire has a stronger association with the children’s adaptive behaviors (particularly social interaction with family or others) when cognition of contingency is low than when it is high. Even though the lower cognition of contingency suggests that it is more difficult for families to show adaptive responses, having a sufficiently great family behavioral repertoire seems to ensure that children with no experience of hikikomori will exhibit adaptive behaviors. In other words, even if the cognitive of contingency or experience frequency is generally low, the family’s functional coping is likely to cause an improvement for individuals with hikikomori if the family acquires a sufficient behavioral repertoire. This result was not found for parents of individuals with hikikomori. This indicates that the family of individuals with hikikomori might not be able to supplement the insufficient cognition of contingency through their behavioral repertoire, even if they have a generally great behavioral repertoire. Therefore, to improve an individual’s hikikomori *via* family support, the family will not only need to acquire a sufficient family behavioral repertoire, but also adequate cognition of contingency and functional family interaction.

Overall, the findings indicate that, although family behavioral repertoire and family interaction are not strongly associated with the expression process of hikikomori (20, 21), they do appear to have strong associations with the improvement process.

## Limitations and Future Directions

There are at least six key limitations of this study. First, we did not consider factors other than family behavioral repertoire and family interaction in relation to the adaptive behaviors of individuals with hikikomori. Family support efforts often target psychological stress and negative evaluations of family members, both of which may strengthen or weaken the influence of family cognitive behavioral factors on the adaptive behaviors of individuals with hikikomori. This point should be examined in future studies. It will be necessary to clarify the best interpretability model by examining a different model from that assumed in this research. Furthermore, although scene experience frequency and cognition of contingency were used as the factors controlling the influence of family interaction, it will be necessary to clarify the factors that moderate the influence of behavioral repertoire and family interaction on adaptive behaviors.

Second, in this study, because we targeted the families of individuals with hikikomori, we cannot deny the possibility of family reporting bias. Specifically, there may be the biases that are caused by recognizing hikikomori positively or negatively (34), and by the influence of the parent’s own psychiatric disorders (22). Therefore, in addition to the factors examined in this study, the evaluation of parents’ own hikikomori and psychiatric disorders should be comprehensively considered in the future. Furthermore, it is possible that families and individuals with hikikomori themselves would focus on different aspects of assessing the associations of family factors with adaptive behaviors. In the future, it will be necessary to confirm whether research on individuals with hikikomori themselves supports the findings of this study. Nevertheless, this study was the first to clarify the family’s cognitive behavioral factors that influence the improvement of individuals with hikikomori, and showed important findings, because the inability to access individuals with hikikomori is the most significant characteristic of the hikikomori case.

Third, we employed a questionnaire study to examine the associations described above. In the future, it will be necessary to examine whether family support targeting family behavioral repertoire and family interaction can actually improve adaptive behaviors.

Fourth, because many in the control group lived separately from their children, these differences between the two groups may have influenced the results. We did not control the difference between living together and separately, because we were examining the influence of parent factors in the hikikomori and control groups. Further studies are needed to examine the influence of demographic variables, including the duration of hikikomori and age, as well as the difference in living arrangements.

Fifth, many of the hikikomori group participants participated in family associations or support centers, suggesting that, to some extent, the family behavioral repertoire and family interactions of the hikikomori group had improved. Therefore, in the future, we should clarify the relationships between thes variables in this study and the severity of an individual’s hikikomori.

Finally, as a secondary purpose to this study, we analyzed both the control and hikikomori groups, and compared both groups preliminarily, but did not compare them directly. It will therefore be necessary to compare both groups directly.

## Ethics Statement

The study was approved by the Waseda University Academic Research Ethical Review Committee (2016–275). We obtained informed consent before conducting the study. In consideration of individuals’ privacy, the study was carried out anonymously.

## Author Contributions

SN and HS designed the study. SN undertook the statistical analysis and wrote the first draft of the manuscript. All the authors have commented on the manuscript. All the authors contributed to and have approved the final manuscript.

## Funding

This work was supported by a JSPS KAKENHI Grant Number JP16J10405, 18H05819 and 19H04882. This project was partially supported by Grant-in-Aid for Scientific Research on Innovative Areas “Will-Dynamics” of The Ministry of Education, Culture, Sports, Science. The funding source had no role in the study design; the collection,analysis, and interpretation of data; the writing of the report; or the decision to submit the article for publication.

## Conflict of Interest

The authors declare that the research was conducted in the absence of any commercial or financial relationships that could be construed as a potential conflict of interest.
