# Supplementary material for: Family Behavioral Repertoires and Family Interaction Influence the Adaptive Behaviors of Individuals With Hikikomori
Source: Front Psychiatry. 2020 Jan 16;10:977. doi: 10.3389/fpsyt.2019.00977 (PMC6976530; doi:10.3389/fpsyt.2019.00977)
Supplement: Supplementary file 1 [file DataSheet_1.docx]

Appendix

Family Interactional Scale for Hikikomori (FIS-H)

| (1) Scene experience frequency | |
| --- | --- |
| 1. | Are told "good morning" by your son/daughter. |
| 2. | Your son/daughter thinks sincerely. |
| 3. | Are told to be "Be quiet" by your son/daughter. |
| 4. | Are told "Thank you" by your son/daughter. |
| 5. | Are told to "Go to hell" by your son/daughter. |
| 6. | Are told "Your food was good" by your son/daughter. |
| 7. | Are told "I got it, I got it" by your son/daughter. |
| 8. | Are told to "Shut up" by your son/daughter. |
| 9. | Your son/daughter expressed his/her honest feelings. |

| (2) Cognition of contingency and family interaction  Cognition of contingency: In general, how likely is this behavior to change? (Questions in each scenes)  Family interaction: In association with your son/daughter, how likely is this behavior to change? (Questions in each scenes) |
| --- |
| Scene 1. Because you were told "I got it, I got it,” you scolded him/her by saying “I don’t like to be spoken to like that.” After that, the number of times to be said "I got it, I got it" is… |
| Scene 2. Because you were told to "Shut up,” you stopped a conversation and left the room. After that, the number of times you were told to "Shut up" is… |
| Scene 3. Though you questioned his/her intentions about the future, because your son/daughter expressed his/her honest feelings, you stopped questioning. After that, the number of times that he/she expresses honest feelings is… |
| Scene 4. Though you talked to him/her with a smile, because you are were told to "Shut up,” you stopped smiling. After that, the number of times you were told to "Shut up" is… |
| Scene 5. Because you were told "Good morning,” you praised him/her by saying “I feel happy when you say that.” After that, the number of times you were told "Good morning" is… |
| Scene 6. Though you were angry and looked angry, because your son/daughter looked thoughtful, you stopped looking angry. After that, the number of times that he/she looked thoughtful is… |
| Scene 7. Because you were told to "Be quiet,” you scolded him/her with “I don’t like to be spoken to like that.” After that, the number of times to be said "Be quiet" is… |
| Scene 8. Because you were told "I got it, I got it,” you looked sad. After that, the number of times you were told to "I got it, I got it" is… |
| Scene 9. Because you were told "Thank you,” you praised “I feel happy when you say that.” After that, the number of times you were told "Thank you" is… |
| Scene 10. Though you angry and looked angry, because your son/daughter said, “Thank you for your concern,” you stopped looking angry. After that, the number of times you were told “Thank you for your concern” is… |
| Scene 11. Though you talked to him/her with a smile, because you were told to "Go to hell,” you stopped smiling. After that, the number of times you were told to "Go to hell" is… |
| Scene 12. Because you were told "Your food is good,” you praised him/her by saying “I feel happy when you say that.” After that, the number of times you were told "Your food is good" is… |
| *Note.* Scene experience frequency items are scored on the following scale: 0: Never, 1: Occasionally, 2: Often, 3: Always. Cognition of contingency and family interaction: 1: Decrease, 2: A little decrease, 3: Unchanged, 4: A little increase, 5: Increase. Items on Scenes 1, 2, 4, 7, 8, and 11 are reverse scored. The questionnaire items are in Japanese, which the authors translated into English; these translations are preliminary (21).  Nonaka, Shimada, Sakai. (2019) doi: 10.1111/jpr.12219 |
